# Treatment with Tumor Necrosis Factor-α Inhibitors, History of Allergy, and Hypercalcemia Are Risk Factors of Immune Reconstitution Inflammatory Syndrome in HIV-Negative Pulmonary Tuberculosis Patients

**DOI:** 10.3390/jcm9010096

**Published:** 2019-12-30

**Authors:** Yoshimasa Hachisu, Yasuhiko Koga, Shu Kasama, Kyoichi Kaira, Masakiyo Yatomi, Haruka Aoki-Saito, Hiroaki Tsurumaki, Yosuke Kamide, Noriaki Sunaga, Toshitaka Maeno, Tamotsu Ishizuka, Takeshi Hisada

**Affiliations:** 1 Department of Allergy and Respiratory Medicine, Gunma University Graduate School of Medicine, Gunma 371-8511, Japan; yhachisu2002@yahoo.co.jp (Y.H.); m09702007@gunma-u.ac.jp (M.Y.); a-haruka@gunma-u.ac.jp (H.A.-S.); m12702056@gunma-u.ac.jp (H.T.); nsunaga@gunma-u.ac.jp (N.S.); mutoyu03@gunma-u.ac.jp (T.M.); 2 Maebashi Red Cross Hospital, Gunma 371-0813, Japan; 3 Institute for Clinical and Translational Science, Nara Medical University Hospital, Nara 634-8522, Japan; s-kasama@bay.wind.ne.jp; 4 Department of Respiratory Medicine, Comprehensive Cancer Center, International Medical Center, Saitama Medical University, Saitama 350-1298, Japan; kkaira1970@yahoo.co.jp; 5 Clinical Research Center for Allergy and Rheumatology. Sagamihara National Hospital, Kanagawa 252-0392, Japan; m08702012@gunma-u.ac.jp; 6 Third Department of Internal Medicine, Faculty of Medical Sciences, University of Fukui, Fukui 910-1193, Japan; tamotsui@u-fukui.ac.jp; 7 Gunma University Graduate School of Health Sciences, Gunma 371-8514, Japan; hisadat@gunma-u.ac.jp

**Keywords:** TNF-α inhibitor, TNFI, tuberculosis, paradoxical response, immune reconstitution inflammatory syndrome, IRIS, history of allergy, hypercalcemia, Th1, Th2

## Abstract

Immune reconstitution inflammatory syndrome (IRIS) is an immune reaction that occurs along with the recovery of the patient’s immunity. Tuberculosis-related IRIS (TB-IRIS) upon tumor necrosis factor (TNF)-α inhibitor treatment has been reported in non-human immunodeficiency virus (HIV) patients. However, the importance of biological treatment, as a risk factor of IRIS, has not yet been established. In this study, we examined TB-IRIS in non-HIV patients to explore the role of TNF-α inhibitor treatment. Out of 188 patients with pulmonary TB, seven patients had IRIS. We examined univariate logistic and multivariate analysis to elucidate risk factors of TB-IRIS. Univariate analysis indicated that usage of immunosuppressive drugs, TNF-α inhibitors, and history of food or drug allergy were significantly related with TB-IRIS. On initial treatment, the values of serological markers such as serum albumin and serum calcium were significantly related with TB-IRIS. There was a higher mortality rate in patients with TB-IRIS. Furthermore, multivariate analysis revealed that usage of TNF-α inhibitors, history of allergy, and serum hypercalcemia were related to TB-IRIS. Usage of TNF-α inhibitors, history of allergy, and serum hypercalcemia may be independent predictors of TB-IRIS in non-HIV patients. Since higher mortality has been reported for TB-IRIS, we should pay attention to TB patients with these risk factors.

## 1. Introduction

Immune reconstitution inflammatory syndrome (IRIS) is well-known as an initial exacerbation after tuberculosis (TB) treatment. It is defined as the deterioration of disease despite appropriate treatment against sensitive *Mycobacterium tuberculosis* and is an immune reaction that occurs with the recovery of the patient’s immunity [1]. In addition to TB, IRIS occurs in infection with cytomegalovirus or cryptococcus [2]. Tuberculosis-related IRIS (TB-IRIS) is reported to occur in 2–25% of human immunodeficiency virus (HIV)-negative pulmonary TB patients [1,3,4,5], and it often occurs during highly active antiretroviral therapy in human HIV-positive patients [6,7]. The development of IRIS is related to mortality rate within 48 weeks after TB treatment [8].

Risk factors of IRIS in a patient undergoing treatment with tumor necrosis factor (TNF)-α inhibitors (TNFIs) are disseminated TB, history of TB, and use of steroids at diagnosis [9,10]. In the TB patient without HIV infection it has been reported that IRIS is not related to the immunosuppressed state [11]. While neutropenic patients or organ transplant recipients have increased risk of IRIS [2], the immunosuppressed state poses a lower risk to IRIS [12] in non-HIV patients. However, whether the use of TNFIs is significantly related to the IRIS development as compared to the patients without TNFI treatment has not yet been elucidated. In this study, we examined the cases of pulmonary TB and examined the frequency and the risk factors of IRIS, and the effect of IRIS on the mortality in non-HIV patients.

## 2. Methods

### 2.1. Study Population

A total of 201 patients were enrolled in this study from amongst the pulmonary TB patients without HIV infection consecutively treated with anti-tuberculosis therapy in our hospital from January 2005 to December 2016. Pulmonary TB was diagnosed by the appearance of infiltrates or consolidates in the radiological examination and the presence of tubercle bacilli in the sputum. This study was conducted with the approval of the Ethics Review Committee of Gunma University Hospital, No. 2017-026.

### 2.2. Diagnosis of IRIS

Immune reconstitution inflammatory syndrome was defined as the deterioration of the existing lesion or appearance of a new lesion in the chest radiological examination despite appropriate anti-tuberculosis therapy performed for more than two weeks [3]. We defined the IRIS-positive group after confirming the IRIS condition according to strict criteria as shown in Table 1 [11] and excluding the complications of other disease, worsening pulmonary shadows, non-sensitivity to initial treatment, and the poor compliance with anti-tuberculosis therapy. We evaluated various factors related to the development of IRIS and examined the association of IRIS on the total mortality during TB treatment. Past TB infection was included in the latent tuberculosis infection (LTBI). Corticosteroids, biological drugs, anti-metabolites, and calcineurin inhibitors were included as immunosuppressive drugs.

### 2.3. Statistical Analysis

For each factor of the IRIS-positive group and the IRIS negative-group, the number of cases and the ratio were calculated on the nominal and average scale, and the standard deviation was calculated on the order scale. Using a logistic regression model for each factor in the presence or absence of IRIS as a dependent variable, univariate analysis was performed to calculate the odds ratio (OR) and 95% confidence interval (CI). Multivariate analysis was performed for the factors with significant differences in univariate analysis.

## 3. Results

**Figure 1:**
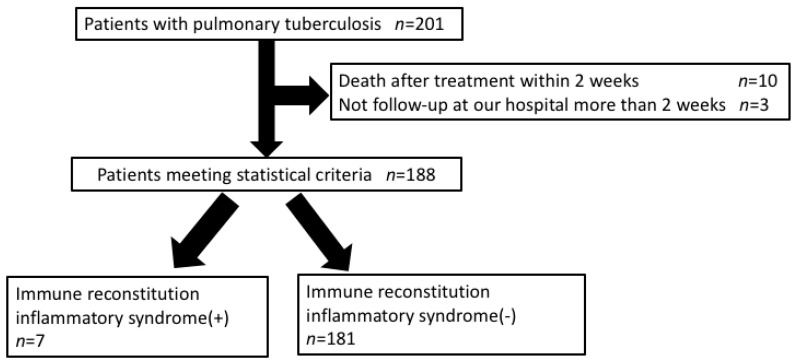
Study population.

Of the consecutive 201 patients with pulmonary TB without HIV infection, 188 patients were enrolled in this study. Ten patients died within two weeks after TB treatment and three patients skipped follow-up for more than two weeks. In this study, seven patients (3.7%) had IRIS (Figure 1).

### 3.1. Association of Patient Background with IRIS Development

As shown in Table 2, there was no significant difference in the age, sex, body mass index (BMI), preference of alcohol, race, history of *M. tuberculosis* infection, and smoking history between IRIS-positive and negative patients. Immunosuppressive drugs were used in 57.1% (four patients) IRIS-positive and 9.4% (17 patients) IRIS-negative patients, and the usage of immunosuppressive drug was found to be significantly associated with the onset of IRIS (*p* = 0.002, (OR) (95% CI) 12.90 (2.65–62.30)). The usage of TNF-α inhibitors (TNFIs) as a biological drug was found to be significantly associated with the onset of IRIS in 28.6% (two patients) IRIS-positive and 1.1% (two patients) IRIS-negative patients (*p* = 0.001, OR (95%CI) 35.80 (4.16–308.0)). Although immunosuppressive drugs, except biological drugs, were used in 28.6% (two patients) of IRIS-positive and 8.8% (16 patients) of IRIS-negative patients, they were not significantly related with the onset of IRIS (*p* = 0.106, OR (95% CI) 4.12 (0.74–23.00)). Diabetes mellitus (DM), renal dialysis, > 70 years of age at the initial treatment, and disseminated TB were not related to the development of IRIS. History of the malignant tumor was not found in the IRIS-positive patients and was not associated with the development of IRIS. There was a significant difference in the presence of food or drug allergy between IRIS-positive (three patients, 42.9%) and IRIS-negative (14 patients, 7.7%) patients (*p* = 0.007, OR (95%CI) 8.95 (1.82–44.00)), as shown in Table 2.

### 3.2. Association of Clinical Parameters with IRIS Development

As shown in Table 3 in the values of serological markers at initial TB treatment, serum albumin showed a significant difference, at 2.44 ± 0.72 g/dL in the IRIS-positive and 3.28 ± 0.80 g/dL in the IRIS-negative patients (*p* = 0.016, OR (95%CI) 0.26 (0.09–0.78)). The serum calcium value corrected by serum albumin value was 10.38 ± 0.86 mg/dL in the IRIS-positive and 9.72 ± 0.68 mg/dL in the IRIS-negative patients, and showed a significant difference (*p* = 0.039, OR (95%CI) 2.38 (1.04–5.44)). There was no significant difference in the other serological markers. Comparison of the periods until three consecutive negative conversions of sputum smears of *M. tuberculosis* showed values of 7.00 ± 4.24 weeks in the IRIS-positive group and 8.03 ± 6.31 weeks in the IRIS-negative group, and no significant difference was found (*p* = 0.746, OR (95% CI) 0.97 (0.80–1.17)). Of the 188 patients, three patients (42.9%) in the IRIS-positive group and 14 patients (7.7%) in the IRIS-negative group died, and the mortality was significantly related with the onset of IRIS (*p* = 0.007, OR (95%CI) 8.95 (1.82–44.00).

### 3.3. Assessment by Multivariate Analysis of IRIS Development

Multivariate analysis was additionally performed for values with significant differences in the univariate analysis. Spearman’s correlation coefficient revealed that serum albumin value correlated with the serum calcium value (correlation coefficients −0.472 and *p* value < 0.001). Fisher’s exact test also revealed that usage of immunosuppressive drugs correlated with history of allergy (*p* = 0.027) or usage of biological drugs (*p* < 0.001). A one-way analysis of variance (ANOVA) between the usage of immunosuppressive drugs and serum calcium value showed a correlation with IRIS onset (*p* = 0.001).

Excluding these factors to avoid correlation, stepwise forward multiple regression analysis of the relationship between IRIS and these factors revealed a significant correlation; the most important factor causing IRIS was the usage of TNF-α (*p* = 0.001) along with history of allergy (*p* = 0.035), and serum calcium value (*p* = 0.024) (Table 4).

Because a small number of IRIS events occurred in this study, we evaluated several models of multivariate analysis predicting IRIS development [13,14]. In analyzed multivariate models, use of TNFI, history of allergy, and hypercalcemia were also important for predicting IRIS development (Table 5).

In this study, four patients were treated with TNFI before TB diagnosis. The characteristics of the patients treated with TNFI are shown in Table 6. The TNFI treatment was terminated in two patients, while it was continued in two patients during TB treatment.

## 4. Discussion

In this study, the usage of TNF-α inhibitors and history of allergy were significantly associated with IRIS development, while the history of TB, tumors, renal dialysis, immunosuppressive drugs except for biological agents, DM, aging, or disseminated TB were not associated with IRIS development. To our knowledge, this is the first study showing the usage of biological drugs, history of allergy, and serum hypercalcemia as a risk factor of IRIS development in pulmonary TB without HIV infection.

In HIV-positive patients, high levels of D-dimer [15], a low number of CD4-positive T cells, high dose of HIV virus, low body mass index, and sputum smear-positive pulmonary TB [8] have been known as risk factors of IRIS. In non-HIV patients, infection, anemia, hypoalbuminemia, low serum lymphocytes [3], elevated eosinophil counts, and low total protein in the pleural effusion in tuberculous pleuritis [16] have also been reported as risk factors of IRIS. Although it has been known that HIV infection is involved with the onset of IRIS, Brown et al. reported that immunosuppressive condition decreased the odds ratio of IRIS onset in HIV-negative cases [12]. In recent years, case reports on TB-IRIS upon treatment with anti-TNF-α antibodies have been increased in HIV-negative cases [10,17,18,19]. In the analysis of patients using anti-TNF-α antibodies against underlying diseases such as rheumatoid arthritis (RA) or Crohn’s disease, disseminated TB, past TB infection, and corticosteroid use at the time of diagnosis have been reported as the risk factors of IRIS development [9]. However, there is no report describing the significance of TNFI treatment on IRIS development. In this study, TNFIs were associated with IRIS development, except the immunosuppressive drugs without TNFIs. Interestingly, RA and Crohn’s disease are Th1 cell-dominant diseases [20,21,22,23], and TNFI promotes the Th2 cytokine-dominant balance via inhibition of TNF-α. The Th2 cytokine-dominant balance caused by TNFI at the initial treatment of TB may be partly associated with IRIS development.

**Figure 2:**
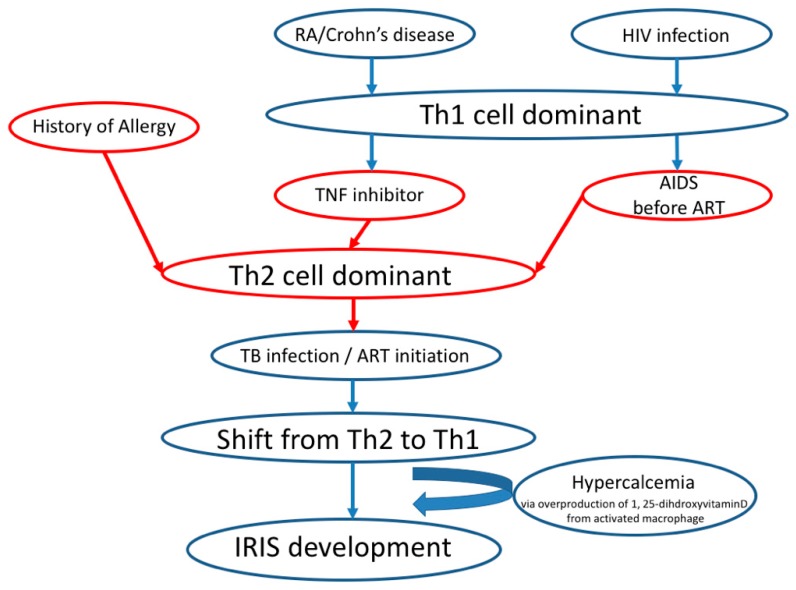
Proposed mechanism from Th2 to Th1 cytokine-dominant balance in IRIS development.

In this study, a significant relationship was found between IRIS development and history of allergy. Most of the food allergies and a part of drug allergies are thought to be an immunoglobulin (IgE)-mediated, Th2 cell-dominant immune response. Initiation of antiretroviral therapy (ART) for HIV patients is associated with a shift from the Th2 to Th1 cell balance, together with IRIS development [24,25]. Patients with a history of allergy or TNFI treatment and patients with the progression of HIV infection to acquired immune deficiency syndrome (AIDS) before ART [26] are in Th2 cytokine-dominant balance. Recently, it has been reported that TNFI treatment for RA deteriorated asthma symptoms, while benralizumab, an anti-IL-5 receptor antagonist, worsened arthritis in a patient with RA-complicated with asthma [27]. This case report indicates that the blockage of the Th1-dominant balance by TNFI treatment promotes the Th1 to Th2 shift and worsens the Th2-dominant disorder, and the inhibition of Th2-dominant balance by IL-5 antagonist initiates Th2 to Th1 shift and deteriorate Th1 dominant disease. There is a possibility that the shift from the Th2 cell cytokine-dominant balance to the Th1 cell cytokine-dominant balance caused by TB infection in a patient with a history of allergy may have influenced IRIS development (Figure 2). Further studies are required to elucidate the mechanism underlying the development of IRIS, which is caused by Th1 cell-dominant balance [2], from the Th2 cell-dominant balance in TB patients with TNFI treatment or history of allergy.

High levels of serum calcium were a risk factor for IRIS development in this study. Consistently, hypercalcemia related to IRIS is known to be caused by overproduction of 1, 25-dihdroxyvitamin D3 (1, 25[OH_2_]D3) secreted from disease-activated macrophages together with Th1 cytokines, e.g., interferon-gamma (IFN-γ) and granuloma formation [28]. The Th1-driven immune responses are thought to be essential for IRIS development in HIV-infected and uninfected patients [2,28].

While the prognosis of TB is relatively good and ≥95% patients may have improved health condition [29], the TB mortality rate was reportedly 12.3% in the culture-positive group [30], and 10.5% in pulmonary TB group [31]. Diabetes mellitus, sputum smear-positive TB, anemia, smoking, and drug-induced hepatitis increased the mortality rate [31]. Erbes et al. reported 25.9% mortality for TB patients cared for in the intensive care unit [32]. Systemic steroid administration was found to be effective in severe IRIS cases [33]. In this study, the mortality rate was higher in the IRIS group (42.9%) than in the non-IRIS group (7.2%), consistent with the previous report [11,34], and patients who developed IRIS should be careful while undergoing treatment with steroid.

There are several limitations in this study. Only 188 patients in a single center were examined, which is a small number. The history of allergy, smoking history, and alcohol intake history was self-reported without any means of validation, and the extent of the history of allergy is unclear. In this study, the history of allergy was diagnosed either as a food or drug allergy by self-report. Therefore, the severity of the allergy remains unknown.

## 5. Conclusions

We demonstrated the possibility of the involvement of TNF-α inhibitors, history of allergy, and hypercalcemia in the development of IRIS in non-HIV pulmonary TB patients. Immune reconstitution inflammatory syndrome is also suggested to be strongly associated with mortality, and patients with these risk factors should be treated carefully.

## Figures and Tables

**Table 1:** Diagnosis of immune reconstitution inflammatory syndrome (IRIS) with fulfilment of the four following criteria. TB: Tuberculosis.

(1) Initial improvement after anti-TB treatment initiation
(2) Worsening of the initial symptoms or onset of new TB-like symptoms after the initiation of anti-TB treatment
(3) Absence of persistently active TB
(4) Absence of any other explanation of clinical deterioration

**Table 2:** Background factors with or without immune reconstitution inflammatory syndrome (IRIS).

Background Factors	All Patients (*n* = 188)	IRIS(+) (*n* = 7)	IRIS(−) (*n* = 181)	*p* Value
Age	62.11 ± 21.54	62.71 ± 23.73	62.08 ± 21.52	0.939
Male	62.8% (118)	57.1% (4)	63.0% (114)	0.754
BMI	19.77 ± 3.58	19.95 ± 5.98	19.76 ± 3.49	0.898
Allergy	9.0% (17)	42.9% (3)	7.7% (14)	0.007 *
Smoking	42.6% (80)	42.9% (3)	42.5% (77)	0.987
Alcohol	33.5% (63)	42.9% (3)	33.1% (60)	0.596
Past infection	14.9% (28)	28.6% (2)	14.4% (26)	0.314
Foreign nationality	13.3% (25)	0.0% (0)	13.8% (25)	0.994
Smear positive	77.1% (145)	85.7% (6)	76.8% (139)	0.602
HIV Infection	0.0% (0)	0.0% (0)	0.0% (0)	-
Immunosuppressiye drugs	11.2% (21)	57.1% (4)	9.4% (17)	0.002 *
Biological drug	2.1% (4)	28.6% (2)	1.1% (2)	0.001 *
Non-biological drug	9.6% (18)	28.6% (2)	8.8% (16)	0.106
Diabetes mellitus	20.7% (39)	28.6% (2)	20.4% (37)	0.605
Dialysis	6.4% (12)	14.3% (1)	6.1% (11)	0.400
Past tumor	19.7% (37)	0.0% (0)	20.4% (37)	0.993
Over 70 years	45.7% (86)	57.1% (4)	45.3% (82)	0.541
Complication of miliary TB	10.1% (19)	28.6% (2)	9.4% (17)	0.123

Values are mean ± SD, or percentage (%) and number. * *p* < 0.01. BMI: body mass index, HIV: human immunodeficiency virus, TB: tuberculosis. Immunosuppressive drugs include prednisolone, calcineurin inhibitors, antimetabolites, and biological drugs.

**Table 3:** Laboratory findings with or without immune reconstitution inflammatory syndrome.

Serological Markers	All Patients (*n* = 188)	IRIS(+) (*n* = 7)	IRIS(−) (*n* = 181)	*p* Value
WBC (/μL)	7075 ± 3232	6700 ± 4125	7090 ± 3206	0.752
Lym (/μL)	988 ± 502	614 ± 359	1003 ± 502	0.053
Hb (g/dL)	11.6 ± 2.0	11.2 ± 2.7	11.7 ± 1.9	0.503
Alb (g/dL)	3.25 ± 0.82	2.44 ± 0.72	3.28 ± 0.80	0.016 *
LDH (U/L)	220 ± 80	203 ± 54	221 ± 81	0.556
ALP (U/L)	293 ± 140	372 ± 316	290 ± 129	0.146
ESR (mm/H)	61.7 ± 33.4	79.1 ± 47.7	61.0 ± 32.6	0.167
CRP (mg/dL)	3.96 ± 4.61	5.83 ± 4.06	3.89 ± 4.62	0.281
Ca (mg/dL)	9.74 ± 0.69	10.38 ± 0.86	9.72 ± 0.68	0.039 *
D-dimer (μg/mL)	6.35 ± 9.51	9.58 ± 14.06	6.19 ± 9.29	0.445
HbA1c (NGSP) (%)	6.22 ± 1.45	5.70 ± 0.50	6.26 ± 1.49	0.292
Treatment course				
Weeks until 3 consecutive smears negative	8.00 ± 6.24	7.00 ± 4.24	8.03 ± 6.31	0.746
Death	9.0% (17)	42.9% (3)	7.7% (14)	0.007 **

Values are mean ± SD, or percentage (%) and number. * *p* < 0.05, ** *p* < 0.01. WBC: white blood cell count. Lym: lymphocyte count, Hb: hemoglobin, Alb: albumin, LDH: lactate dehydrogenase, ALP: alkaline phosphatase, ESR: erythrocyte sedimentation rate, CRP: C-reactive protein, Ca: calcium.

**Table 4:** Univariate or multivariate analysis for IRIS development.

	Univariate	Multivariate	Wald	*p* Value
Background factors				
Age	1.00 (0.97–1.04)			
Male	0.78 (0.17–3.61)			
Body mass index	1.01 (0.81–1.27)			
Allergy	8.95 (1.82–44.00)	10.39 (1.17–91.88)	4.43	0.035 *
Smoking	1.01 (0.22–4.66)			
Alcohol	1.51 (0.33–6.98)			
Past infection	2.38 (0.44–12.90)			
Immunosuppressive drug	12.90 (2.65–62.30)			
Biological drug	35.80 (4.16–308.00)	142.65 (6.87–2962.35)	10.27	0.001 **
Non-biological immunosuppressive drug	4.12 (0.74–23.00)			
Diabetes mellitus	1.56 (0.29–8.35)			
Dialysis	2.58 (0.29–23.30)			
Complication of miliary TB	3.86 (0.70–21.40)			
Serological markers				
White blood cell count (/μL)	1.00 (1.00–1.00)			
Lymphocyte count (/μL)	1.00 (1.00–1.00)			
Albumin (g/dL)	0.26 (0.09–0.78)			
ESR (mm/H)	1.02 (0.99–1.04)			
C-reactive protein (mg/dL)	1.07 (0.94–1.22)			
Calcium (mg/dL)	2.38 (1.04–5.44)	5.82 (1.26–26.92)	5.07	0.024 *
HbA1c (%)	0.54 (0.17–1.70)			
Treatment course				
Death	8.95 (1.82–44.00)			

Values are odds ratio and 95% confidence interval. * *p* < 0.05, ** *p* <0.01. TB: tuberculosis, ESR: erythrocyte sedimentation rate.

**Table 5:** Multivariate predictors of IRIS development.

	Multivariate1	*p* Value	Multivariate2	*p* Value	Multivariate3	*p* Value
Background factors						
Allergy	9.01 (1.54–52.80)	0.015 *	9.96 (1.72–57.90)	0.010 *		
Biological drug	36.10 (3.39–385.0)	0.003 **			98.2 (6.79–1420)	<0.001 **
Serological markers						
Calcium (mg/dL)			2.36 (1.01–5.53)	0.049 *	2.85 (1.15–7.10)	0.024 *

Values are odds ratio and 95% confidence interval. * *p* < 0.05, ** *p* < 0.01.

**Table 6:** Patient characteristics treated with anti-tumor necrosis factor-α antibodies.

Patient No	Age	sex	Extrapulmonary Tuberculosis	Underlying Disease	Anti-TNFa Regimen	Using Time (months)	IRIS	Discontinuation of Biological Drugs
1	68	F	Miliary TB	RA	Adalimumab	48	No	Discontinuation
2	58	M	None	Crohn’s disease	Adalimumab	14	Yes	Discontinuation
3	75	M	None	Psoriasis vulgaris	Adalimumab	24	No	Continuation
4	36	M	None	Crohn’s disease	Infliximab	64	yes	Continuation

RA: Rheumatoid arthritis.
